# Functional Tolerance is Maintained Despite Proliferation of CD4 T Cells after Encounter with Tissue-derived Antigen

**DOI:** 10.1080/1044667031000137656

**Published:** 2002-09

**Authors:** Lara J. Ausubel, Anna Chodos, Nyree Bekarian, Abul K. Abbas, Lucy S. K. Walker

**Affiliations:** Department of Pathology University of California San Francisco 513 Parnassus Avenue San Francisco CA 94143 USA

## Abstract

Since negative selection in the thymus is incomplete, some self-reactive T cells are able to mature and seed the periphery. To study how these T cells interact following encounter with the self-protein they recognize in the periphery, we have developed an adoptive transfer system in which HEL-specific TCR transgenic CD4 T cells are transferred to mice expressing HEL protein in the pancreas under the control of the rat insulin promoter. Here we show that after adoptive transfer of HEL-specific T cells functional tolerance is maintained despite evidence that the T cells encounter and respond to pancreas-expressed antigen. Even the provision of an additional activation stimulus by peripheral immunization with HEL protein is insufficient to induce the T cells to cause autoimmune tissue injury. However, in the presence of blocking anti-CTLA-4-mAb, immunized adoptive transfer recipients rapidly developed diabetes. These data suggest that the CTLA-4 pathway regulates the pathogenicity of antigen-specific T cells following a peripheral activation stimulus.


*Developmental Immunology*, September 2002, Vol. 9 (3), pp. 173–176

Taylor & Francis

Taylor & Francis Group

### Functional Tolerance is Maintained Despite Proliferation of CD4 T Cells after Encounter with Tissue-derived Antigen*

LARA J. AUSUBEL, ANNA CHODOS, NYREE BEKARIAN, ABUL K. ABBAS and LUCY S.K. WALKER†

*Department of Pathology, University of California San Francisco, 513 Parnassus Avenue, San Francisco, CA 94143, USA*

Since negative selection in the thymus is incomplete, some self-reactive T cells are able to mature and seed the periphery. To study how these T cells interact following encounter with the self-protein they recognize in the periphery, we have developed an adoptive transfer system in which HEL-specific TCR transgenic CD4 T cells are transferred to mice expressing HEL protein in the pancreas under the control of the rat insulin promoter. Here we show that after adoptive transfer of HEL-specific T cells functional tolerance is maintained despite evidence that the T cells encounter and respond to pancreas-expressed antigen. Even the provision of an additional activation stimulus by peripheral immunization with HEL protein is insufficient to induce the T cells to cause autoimmune tissue injury. However, in the presence of blocking anti-CTLA-4-mAb, immunized adoptive transfer recipients rapidly developed diabetes. These data suggest that the CTLA-4 pathway regulates the pathogenicity of antigen-specific T cells following a peripheral activation stimulus.

*Keywords*: Tolerance; CTLA-4; Diabetes; Transgenic mice

### INTRODUCTION

Deletion of self-reactive T cells during thymic development is not comprehensive and some such cells are able to complete their maturation and populate the periphery. Clearly, in normal individuals, the presence of self-reactive T cells does not cause autoimmune disease. In some circumstances, the lack of pathogenicity may be a result of T cell ignorance, perhaps because the relevant self-antigen is sequestered in locations that are inaccessible to immune surveillance. An alternative possibility is that self-reactive T cells interact with self-antigen in the periphery, but that this interaction does not cause the T cells to develop into pathogenic effector cells.

One gene that has been implicated in the induction and/or maintenance of peripheral T cell tolerance is CTLA-4. Blocking the CTLA-4 pathway by administration of monoclonal antibody interferes with tolerance induction to intravenous soluble antigen (Perez *et al.*, 1997) and to superantigens (Walunas and Bluestone, 1998). In addition, CTLA-4 blockade has been shown to exacerbate autoimmune disease in a number of murine models (Karandikar *et al.*, 1996; Luhder *et al.*, 1998). A role for CTLA-4 in controlling tolerance receives additional support from the observation that polymorphisms in the region where the CTLA-4 gene is located have been linked to the occurrence of autoimmunity in humans (Kristiansen *et al.*, 2000; Rodriguez *et al.*, 2002).

In this study we have set out to model how self-reactive CD4 T cells respond to encounter with self-antigen expressed in a peripheral tissue. To this end, we have developed an adoptive transfer system in which HEL-specific CD4 T cells (3A9) from a TCR transgenic mouse are introduced into mice expressing HEL under the control of the rat insulin promoter in the pancreas (RIP-HEL mice). The adoptive transfer system allows us to exclude the effects of central tolerance and, instead, to focus on the peripheral tolerance mechanisms that control self-reactive T cells that have escaped thymic deletion. Using a clonotypic antibody to identify the antigen-specific T cells we show that naïve T cells gain access to self-antigen expressed in the pancreas, but despite this, the T cells remain functionally tolerant and fail to induce diabetes. In fact, even after activation in the periphery by immunization with HEL protein, 3A9 T cells do not have pathogenic effects unless the CTLA-4 pathway is blocked. These data suggest that recognition of self-antigen by self-reactive T cells is not sufficient to trigger pathogenicity, but that after immunization the CTLA-4

*Presented at the Proceedings of the 4th Germinal Center Conference, Groningen, The Netherlands, June 2002.

†Corresponding author. Address: MRC Centre for Immune Regulation, University of Birmingham Medical School, Vincent Drive, Birmingham, B15 2TT, UK. Tel.: +44 (0) 121 414 6854. Fax: +44 (0) 121 414 3599. E-mail: L.S. Walker@bham.ac.uk

ISSN 1044-6672 print © 2002 Taylor & Francis Ltd
DOI: 10.1080/1044667031000137656

pathway is required to prevent the onset of autoimmune tissue destruction.

### RESULTS

### HEL-specific CD4 T Cells Transferred to RIP-HEL Recipients Fail to Induce Diabetes unless Previously Activated *In Vitro*

To study the regulation of CD4 T cells that escape central tolerance and populate the periphery, we used an adoptive transfer system in which naïve HEL-specific 3A9 T cells were introduced into mice bearing HEL as a self-protein in the pancreas (RIP-HEL mice). Introduction of naïve 3A9 T cells did not trigger diabetes induction in RIP-HEL recipients as evidenced by the maintenance of stable blood glucose levels (1) . Recipient mice were monitored for up to 6 weeks with no evidence of increased blood glucose levels (data not shown). To assess whether prior activation of the 3A9 T cells was sufficient to convert them into pathogenic effector cells, naïve 3A9 T cells were activated *in vitro* with $HEL_{46-61}$ peptide and APCs for 4 days prior to adoptive transfer. The activated cells that were recovered from these cultures were capable of rapid diabetes induction after adoptive transfer into RIP-HEL recipients (Fig. 1).

### *Ex Vivo* Proliferative Responses Suggest Transferred HEL-specific T Cells are not Ignorant of Pancreatic HEL Protein

If the lack of diabetes induction after transfer of naïve T cells was due to ignorance of pancreatic HEL, then 3A9 T cells should behave similarly regardless of whether they are transferred to RIP-HEL mice or to non-transgenic control mice. To test this premise, 3A9 T cells were transferred to RIP-HEL mice or littermate controls, left for 6 days, then peripheral lymph nodes of recipients were isolated and the *in vitro* proliferative response to $HEL_{46-61}$ peptide assessed. The proliferative response of lymph node cells to HEL peptide was markedly decreased after adoptive transfer of naïve 3A9 T cells to RIP-HEL mice compared to non-transgenic littermates (Fig. 2). This suggests that expression of the HEL protein in the pancreas of RIP-HEL mice has functional consequences for the behavior of transferred 3A9 T cells.

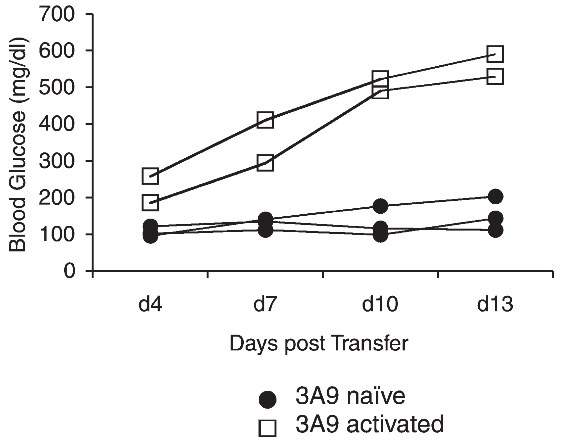

FIGURE 1 Naïve 3A9 T cells fail to induce diabetes upon transfer to RIP-HEL recipients unless previously activated *in vitro*. 5 million naïve or *in vitro* activated 3A9 + T cells were transferred to RIP-HEL mice. Blood glucose readings are shown at the indicated time post adoptive transfer.

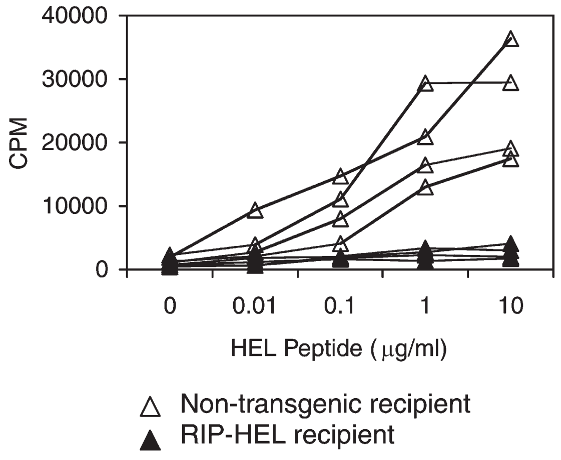

FIGURE 2 Evidence that transferred 3A9 T cells are influenced by pancreatic HEL expression. 2.5 million naïve 1G12 + T cells were adoptively transferred into RIP-HEL mice or non-transgenic littermates. 6 days later peripheral lymph nodes from recipient mice were restimulated *in vitro* with $HEL_{46-61}$ peptide and proliferation assessed by $^3$H-thymidine incorporation 72 h later.

### Transferred HEL-specific T Cells Proliferate Specifically in the Pancreatic Lymph Node

To visualize the response of the transferred 3A9 T cells to pancreatic antigen, carboxy-fluorescein diacetate succinimidyl ester (CFSE) labeling studies were carried out. CFSE-labeled 3A9 T cells were transferred to RIP-HEL mice and at the indicated timepoints the mice were sacrificed and pancreatic and inguinal lymph nodes were removed. Flow cytometric analysis revealed a sequential loss of CFSE dye from the 3A9 cells in the pancreatic LN, but not from the 3A9 cells in the inguinal LN (Fig. 3). This implies that the HEL protein expressed in the pancreas stimulates the proliferation of the 3A9 T cells specifically in the local draining lymph node while 3A9 cells in other lymph nodes remain undivided.

### Wild-type 3A9 Cells Fail to Induce Diabetes in RIP-HEL Mice even after Immunization with HEL Protein, but CTLA-4 Blockade Triggers Aggressive Diabetes

To test whether additional activation signals were required to trigger pathogenic activity in adoptively transferred naïve 3A9 T cells, recipient RIP-HEL mice were immunized intraperitoneally with alum-precipitated HEL protein. Despite increasing the number and activation

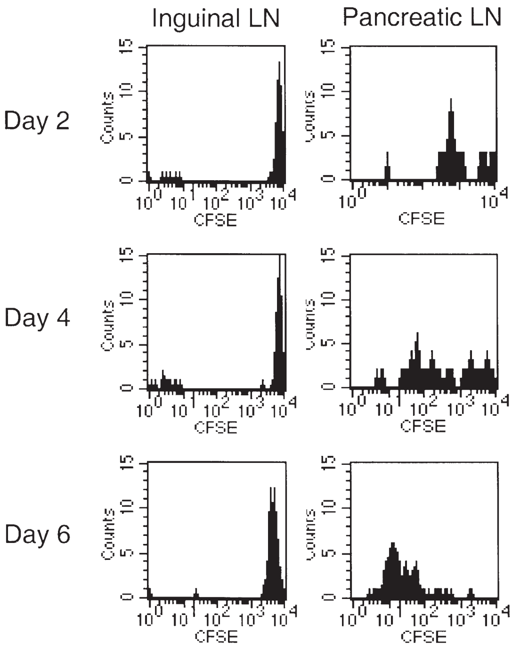

FIGURE 3 Transferred 3A9 T cells proliferate in the pancreatic lymph node of RIP-HEL recipients. 2.5 million naïve 1G12 + T cells were CFSE labeled and adoptively transferred into RIP-HEL recipients. At the indicated time point post transfer, mice were sacrificed and the pancreatic and inguinal lymph nodes dissected and stained with anti-CD4-PerCP and biotinylated 1G12 followed by streptavidin-PE. Histograms show the CFSE profiles of gated CD4 + 3A9 + T cells.

status of the 3A9 T cells (data not shown), the immunization did not trigger diabetes induction. However, if a blocking anti-CTLA-4 antibody was also administered, then diabetes was rapidly induced after i.p. immunization. This suggests that the inability of 3A9 T cells to induce autoimmune tissue destruction in RIP-HEL mice is attributable to the CTLA-4 pathway (Fig. 4).

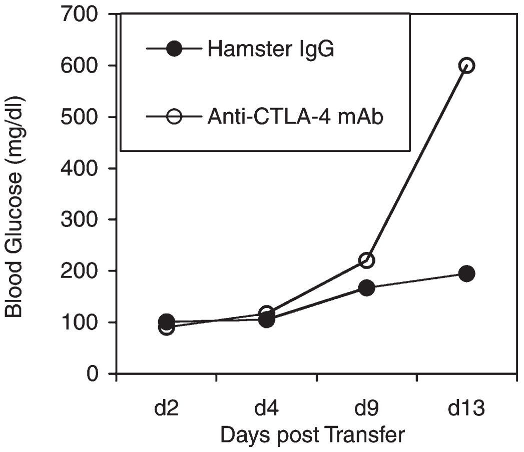

FIGURE 4 Immunization of RIP-HEL recipients of naïve 3A9 T cells fails to trigger diabetes induction unless the CTLA-4 pathway is blocked. 2.5 million 3A9 T cells were transferred to RIP-HEL recipients that were immunized with alum-precipitated HEL protein 24 h later. 100 μg anti-CTLA-4 antibody or control antibody (hamster IgG) was injected intraperitoneally on days 0, 2, 4 and 6.

### DISCUSSION

Self-reactive T cells exist in the peripheral repertoire of normal individuals without triggering overt autoimmunity (Lohmann *et al.*, 1996; Semana *et al.*, 1999). To study how such T cells respond to encounter with peripherally expressed self-protein, we have adoptively transferred HEL-specific T cells into mice expressing HEL protein in the pancreas. Despite bearing high numbers of T cells specific for pancreatic HEL antigen, recipient mice showed no signs of developing diabetes. One potential explanation was that the transferred T cells remained ignorant of pancreatic HEL. However, this was refuted by the observation that the adoptively transferred T cells divided in the pancreatic lymph node.

Despite evidence of a productive encounter with antigen, these T cells do not become pathogenic and fail to trigger diabetes.

The division of transferred T cells in the pancreatic lymph node is consistent with the idea that tissue-derived antigens can be transported to the draining lymph node for presentation to T cells (Hoglund *et al.*, 1999; Sarukhan *et al.*, 1999), most likely by dendritic cells providing surveillance within the tissue. In fact, by day 6 post adoptive transfer, all of the 3A9 T cells in the pancreatic lymph node were CFSE low suggesting that cell division was a universal response to self-antigen, and was not restricted to a sub-population of the transferred cells. 3A9 T cells in lymph nodes that did not drain the antigen-bearing tissue maintained high levels of CFSE, suggesting that divided 3A9 T cells from the pancreatic lymph node did not recirculate through the lymphoid system. We are currently investigating whether the encounter with self-antigen in the pancreatic lymph node is sufficient to trigger migration into the pancreas itself. Even if the cells reach the pancreas, clearly, they are prevented from instigating tissue damage since RIP-HEL recipients of 3A9 T cells remain healthy and exhibit normal glucose homeostasis.

One of the most surprising findings in this study was that peripheral immunization with the relevant antigen in immunogenic form was not sufficient to trigger diabetes in RIP-HEL mice that had received 3A9 T cells. We had predicted that the reason that interaction with pancreas-derived HEL did not cause diabetes was that the antigen-bearing APCs lacked costimulatory ligands. Consistent with a minimal role for costimulation in T proliferation induced by endogenous tissue proteins, the proliferation of CD8 T cells to another pancreas-expressed protein has been shown to be CD28-independent (Hernandez *et al.*, 2001). Since administering the antigen in an immunogenic form would presumably ensure that T cells received costimulation from activated APC, we expected such T cells to be capable of diabetes induction. Interestingly, it appeared that the CTLA-4 pathway prevented this outcome, since diabetes only emerged in the presence of a blocking anti-CTLA-4 antibody in this experiment. These data imply that if self-reactive T cells are activated in the periphery, the CTLA-4 pathway plays a non-redundant role in the prevention of autoimmune tissue damage.

In summary, our model reveals that functional tolerance can be maintained despite the presence of a high precursor frequency of CD4 T cells specific for a pancreatic antigen. Even following activation of such T cells by peripheral immunization, the CTLA-4 pathway provides a second line of defense in the prevention of autoimmune disease. These data shed light on the interaction of naïve T cells with tissue-expressed self-proteins and the conditions under which this can lead to autoimmune disease.

### MATERIALS AND METHODS

### Mice

3A9 TCR transgenic mice were maintained on an MRL background. RIP-HEL ILK mice were obtained from C. Goodnow and bred to MRL. Animals were maintained at UCSF animal facility in accordance with university guidelines and used between 6 and 12 weeks of age. Mice were genotyped using PCR and flow cytometry.

### T Cell Transfers

Combined LN (axillary, inguinal, brachial and mesenteric) from 3A9 mice were stained with the clonotypic Ab, 1G12 and the number of T cells expressing the transgenic TCR was assessed by flow cytometry. The indicated number of 1G12-positive cells was transferred into recipient mice by tail vein injection. Where indicated, cells were incubated prior to transfer with 1 μM CFSE (Molecular Probes, Eugene, OR) for 10 min at room temperature followed by two washes with RPMI supplemented as below. For some experiments, 3A9 cells were activated *in vitro* with 1 μg/ml $HEL_{46-61}$ peptide in complete medium. On day 4, cells were passed over Lympholyte-M (Cedarlane Labs, Ontario, Canada), washed twice, stained with clonotypic antibody and $6\times10^6$ 3A9+ cells were adoptively transferred into RIP-HEL recipients. All antibodies were purchased from Pharmingen unless otherwise indicated.

### *In Vitro* Restimulation

Pooled peripheral LN (inguinal, axillary and brachial) from RIP-HEL or control recipients of 3A9 T cells were cultured at a density of $5\times10^5$ total cells/well in 0.2 ml of RPMI 1640 supplemented with 1 mM L-glutamine, penicillin, streptomycin, non-essential amino acids, sodium pyruvate, HEPES (all from Life Technologies, Grand Island, NY), $5\times10^{-5}$ M 2-ME and 10% FBS (Sigma, St Louis, MO) containing the indicated concentration of $HEL_{46-61}$ peptide. Samples were pulsed with 1 μCi $^3$H-thymidine (New England Nuclear, Boston, MA) for the final 7–8 h of the 72 h period and incorporated radioactivity was measured in a Betaplate scintillation counter (LBK Pharmacia, Piscataway, NJ).

### Immunization

HEL protein (Sigma, St. Louis, MO) was alum-precipitated and 100 μg was administered i.p. 24 h following adoptive transfer of 3A9 T cells. Hamster IgG or anti-CTLA-4- (4F10) were administered intraperitoneally where indicated on days 0, 2, 4 and 6.

### Blood Glucose

Blood glucose levels were measured every 3–4 days (Glucometer Elite XL, Bayer corporation, Elkhart, IN) and mice were considered diabetic following two consecutive readings of >250 mg/dl.

### *Acknowledgements*

We are grateful to C. Goodnow for the ILK mice and to E. Unanue and D. Peterson for providing us with the 1G12 hybridoma and Jeff Bluestone for anti-CTLA-4 antibody. LSKW is funded by The Wellcome Trust. This work was supported by NIH grants PO1-AI35297 and R37-AI 25022 (AKA). We thank J. Cyster and M. Krummel for helpful comments on the manuscript.

### *References*

Hernandez, J., Aung, S., Redmond, W.L. and Sherman, L.A. (2001) "Phenotypic and functional analysis of CD8(+) T cells undergoing peripheral deletion in response to cross-presentation of self-antigen", *J. Exp. Med.* **194**, 707–717.

Hoglund, P., Mintern, J., Waltzinger, C., Heath, W., Benoist, C. and Mathis, D. (1999) "Initiation of autoimmune diabetes by developmentally regulated presentation of islet cell antigens in the pancreatic lymph nodes", *J. Exp. Med.* **189**, 331–339.

Karandikar, N.J., Vanderlugt, C.L., Walunas, T.L., Miller, S.D. and Bluestone, J.A. (1996) "CTLA-4: a negative regulator of autoimmune disease", *J. Exp. Med.* **184**, 783–788.

Kristiansen, O.P., Larsen, Z.M. and Pociot, F. (2000) "CTLA-4 in autoimmune diseases-a general susceptibility gene to autoimmunity?", *Genes Immun.* **1**, 170–184.

Lohmann, T., Leslie, R.D. and Londei, M. (1996) "T cell clones to epitopes of glutamic acid decarboxylase 65 raised from normal subjects and patients with insulin-dependent diabetes", *J. Autoimmun.* **9**, 385–389.

Luhder, F., Hoglund, P., Allison, J.P., Benoist, C. and Mathis, D. (1998) "Cytotoxic T lymphocyte-associated antigen 4 (CTLA-4) regulates the unfolding of autoimmune diabetes", *J. Exp. Med.* **187**, 427–432.

Perez, V.L., van Parijs, L., Biuckians, A., Zheng, X.X., Strom, T.B. and Abbas, A.K. (1997) "Induction of peripheral T cell tolerance *in vivo* requires CTLA-4 engagement", *Immunity* **6**, 411–417.

Rodriguez, M.R., Nunez-Roldan, A., Aguilar, F., Valenzuela, A., Garcia, A. and Gonzalez-Escribano, M.F. (2002) "Association of the CTLA4 3′ untranslated region polymorphism with the susceptibility to rheumatoid arthritis", *Hum. Immunol.* **63**, 76–81.

Sarukhan, A., Lechner, O. and von Boehmer, H. (1999) "Autoimmune insulitis and diabetes in the absence of antigen-specific contact between T cells and islet beta-cells", *Eur. J. Immunol.* **29**, 3410–3416.

Semana, G., Gausling, R., Jackson, R.A. and Hafler, D.A. (1999) "T cell autoreactivity to proinsulin epitopes in diabetic patients and healthy subjects", *J. Autoimmun.* **12**, 259–267.

Walunas, T.L. and Bluestone, J.A. (1998) "CTLA-4 regulates tolerance induction and T cell differentiation *in vivo*", *J. Immunol.* **160**, 3855–3860.

